# SIRT6 Promotes the Progression of Prostate Cancer via Regulating the Wnt/*β*-Catenin Signaling Pathway

**DOI:** 10.1155/2022/2174758

**Published:** 2022-02-25

**Authors:** Xian Zhang, Rong Chen, Li-De Song, Ling-Fei Zhu, Jian-Fei Zhan

**Affiliations:** ^1^Department of Urology Surgery, The Wenzhou Central Hospital, The Dingli Clinical College of Wenzhou Medical University, Wenzhou, Zhejiang, China; ^2^Department of Urology Surgery, The People's Hospital of Zhuji, Shao Xing, Zhejiang, China

## Abstract

Sirtuin 6 (SIRT6), a DNA repair-related gene, has undergone an extremely thorough study for its involvement in the development of many different cancers. The objective of our study was to explore the function and mechanism of SIRT6-induced regulation of prostate cancer (PCa). RT-PCR was performed to validate the levels of SIRT6 in PCa cell lines. Cell proliferation, migration, and invasion of cells with SIRT6 knockdown were assessed using CCK-8 assay, colony formation assay, wound-healing assay, and transwell assay. Western blot was applied to assess the related proteins. We found that SIRT6 expression was distinctly upregulated in PCa specimens and cells. Loss-of-functional assays revealed that SIRT6 silence suppressed the proliferation and metastasis of PCa cells. Mechanistic studies revealed that SIRT6 silence inhibited Wnt/*β*‐catenin signaling and EMT progress. Overall, the study confirmed the upregulation of SIRT6 in patients with PCa and its association with the progression. SIRT6 promoted PCa progression by regulating Wnt/*β*‐catenin signaling, providing a promising biomarker and treatment approach for preventing PCa.

## 1. Introduction

Prostate cancer (PCa) is one of the most common malignancies in elderly males around the world [1]. The incidence rates are increasing over the last two decades. The clinical outcome of many patients with PCa has achieved a positive situation after the application of early surgical excisions [2]. However, a poor prognosis was frequently observed in patients with advanced stages or unresectable tumors [3]. In addition, neoadjuvant chemotherapy and surgery are necessary for the improvement of long-term survivals [4, 5]. Although plenty of effort has been made to improve the treatment efficiency of PCa, the potential mechanisms involved in PCa progression have limited the development of effective treatments.

Sirtuin 6 (SIRT6) is a nicotinamide adenine dinucleotide (NAD+)-dependent histone deacetylase which has been confirmed to remove acetyl groups from histone 3 lysine 9 and histone 3 lysine 56 motifs [6, 7]. More and more evidence have demonstrated that histone deacetylase exhibited multiple effects, such as inhibition of suppression of cellular transformation, maintenance of genome stability, and glucose homeostasis [8, 9]. As a multifunctional nuclear protein, the functions of SIRT6 are complex. It has been demonstrated that SIRT6 exhibited a regulatory effect in several biological progressions, including bone disorders, inflammation, diabetes, heart and liver diseases, neurodegenerative, glucose metabolism, longevity, genome stability, and various tumors [10, 11]. Besides, several research studies have reported that SIRT6 plays an important role in DNA damage, repair, and mutagenesis [12, 13]. In recent years, more and more studies have reported the distinct dysregulation of SIRT6 in many types of tumors [14, 15]. However, the expressing pattern of SIRT6 exhibited a different trend based on the types of tumors. In pancreatic cancer, SIRT6 expression was distinctly decreased and its overexpression suppressed tumor metastasis [16]. However, SIRT6 was found to be overexpressed in diffuse large B-cell lymphoma and promote the proliferation and invasion of tumor cells via mediating PI3K/Akt signaling [17]. The potential function of SIRT6 in PCa remained largely unclear.

In this research, we aimed to examine the expression of SIRT6 in PCa patients, investigate its possible functions, and characterize molecular mechanisms involved in SIRT6 roles in PCa progression. Our findings provided a novel perspective therapeutic target of SIRT6 in PCa.

### 1.1. Patients and Methods

#### 1.1.1. Cell Transfection

A panel of PCa cell lines including PC-3, 22RV1, and DU145 cells and a human normal prostate cell line (WPMY) were all obtained from BeNa Company (Suzhou, Jiangsu, China) and maintained in RPMI-1640 media (Beikai, Changsha, Hunan, China) with 10% FBS. Besides, TransEasy transfection reagent kits (Fujiyin, Chengdu, Sichuan, China) were then applied to perform the cell transfection in accordance with the kits' protocols. The siRNAs (si-NC and si-SIRT6) were purchased from JiMa Biological Corporation (Suzhou, Jiangsu, China).

#### 1.1.2. Real-Time PCR

TRIzol reagents (Qianchen, Pudong, Shanghai, China) were employed to extract total RNAs. The first-strand cDNA synthesis was carried out with a High-Fidelity 1st Strand cDNA synthesis kit (Agilent, Chaoyang, Beijing, China), and the qRT-PCR analysis for SIRT6 detection was carried out by the use of SYBR-Green Real-Time Mix kits (Shenggong, Songjiang, Shanghai, China). The expressing values of SIRT6 were normalized to GAPDH and calculated using the 2^−△△Ct^ method. The PCR primers were designed as follows: SIRT6 forward, 5′-CCCACGGAGTCTGGACCAT-3′ and reverse, 5′-CTCTGCCAGTTTGTCCCTG-3′ and GAPDH forward, 5′-CTGGGCTACACTGGCACC-3′ and reverse, 5′-AAGTGGTCGTTGAGGGCAATG-3′.

#### 1.1.3. Cell Viability Detection

The cell viability was assessed by CCK-8 assays. In short, 2000 PC-3 or DU145 cells after treatment with si-NC were seeded in 96-well plates per well. After culturing for 48 h, 72 h, and 96 h, the cells were treated with CCK8 reagents (10 *μ*l; BOSTER, Wuhan, Hubei, China). Then, the absorbance at 450 nm at the indicated time point was evaluated by a microreader.

#### 1.1.4. Colony Formation Assay

Briefly, PC-3 or DU145 cells after treatment with si-NC were plated into 6-well plates at a density of 500 cells per well. Cells were then cultured for about 2 weeks. Then, paraformaldehyde (4%) (Sigma, Yangfu, Shanghai, China) and crystal violet (0.1%) (Solarbio, Tongzhou, Beijing, China) were applied to fix and stain the cell colonies, respectively. Finally, the cell colonies were observed using a microscope (XHC-BV1; DongFangHuaCe, Chaoyang, Beijing, China).

#### 1.1.5. Wound-Healing Assay

In short, PC-3 or DU145 cells were treated with si-NC. Then, cells were planted into 12-well plates and continued to be cultured until 100% cell confluence. A pipette tip (200 *μ*l) was then employed to generate a wound field. After that, the cells were washed and observed by a microscope at 0 h and 48 h (XHC-BV1; DongFangHuaCe, Chaoyang, Beijing, China).

### 1.2. Transwell Assay

Cellular invasion was evaluated by transwell invasion assays using Corning Costar transwell inserts (Lianshuo Biotech, Qingpu, Shanghai, China). First, the upper chambers of the transwell inserts were treated with Matrigel. Then, PC-3 or DU145 cells after treatment with si-NC were resuspended in serum-free media and then planted into the upper chambers of the inserts. In addition, the lower chambers were loaded with a medium containing 15% FBS. After 24 h, the invaded cells in the lower chamber were fixed in 4% paraformaldehyde and stained with 0.1% crystal violet. Finally, these cells were observed and photographed under an inverted microscope (XHC-BV1; DongFangHuaCe, Chaoyang, Beijing, China).

### 1.3. Western Blot

In brief, the PC-3 or DU145 cells were lysed using a cell lysates extraction kit (X-Y Bioscience, Minhang, Shanghai, China), and the lysates were quantified by a BCA kit (Jingke Chemical Technology, Jinshan, Shanghai, China). Subsequently, 20 *μ*g of the protein sample was fractionated by 10% SDS-PAGE, which was then transferred to PVDF membranes (Millipore, Bedford, Massachusetts, USA). Proteins were blocked by 5% skim milk and then were examined by corresponding antibodies using a super-enhanced ECL detection kit (Servicebio, Wuhan, Hubei, China). The primary antibodies against *β*-catenin, cyclin D1, and c-myc were purchased from Wuhan BOSTER Co., Ltd. (Wuhan, Hubei, China).

### 1.4. Statistical Analysis

Data analysis was performed using SPSS 19.0 statistical software (SPSS, Chicago, IL, USA). The Student's *t*-test was applied to two-group analysis. A value of *p* < 0.05 was considered to indicate statistical significance.

## 2. Results

### 2.1. Upregulation of SIRT6 in PCa Tissues and Cell Lines

**Figure 1:**
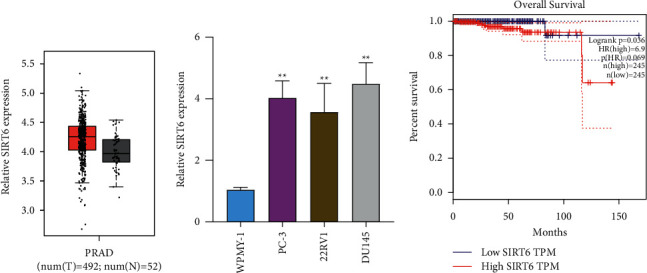
SIRT6 was upregulated in PCa specimens and cell lines. (a) GEPIA was used to analyze the expression of SIRT6 in PCa specimens (*n* = 492) and nontumor specimens (*n* = 52) based on TCGA data sets. (b) qPCR detected the relative SIRT6 levels in PCa cells. (c) Survival value of SIRT6 expression in PCa patients based on TCGA data sets. ^*∗∗*^*p* < 0.01.

To explore the possible function of SIRT6 in PCa, we searched “GEPIA”, which can be used to analyze the expressions of various genes in tumors based on TCGA data sets [18], finding that SIRT6 expression was distinctly upregulated in PCa specimens compared with normal specimens (*p* < 0.01, Figure 1(a)). Moreover, the levels of SIRT6 in three PCa cell lines were also higher than those in WPMY-1 (Figure 1(b)). In addition, survival assays revealed that high SIRT6 expression was associated with a shorter overall survival of PCa patients (Figure 1(c)). Overall, our findings revealed SIRT6 as a possible regulator in the progression of PCa.

### 2.2. Overexpression of SIRT6 Contributed to the Inhibition of Cellular Proliferation in PCa Cells

**Figure 2:**
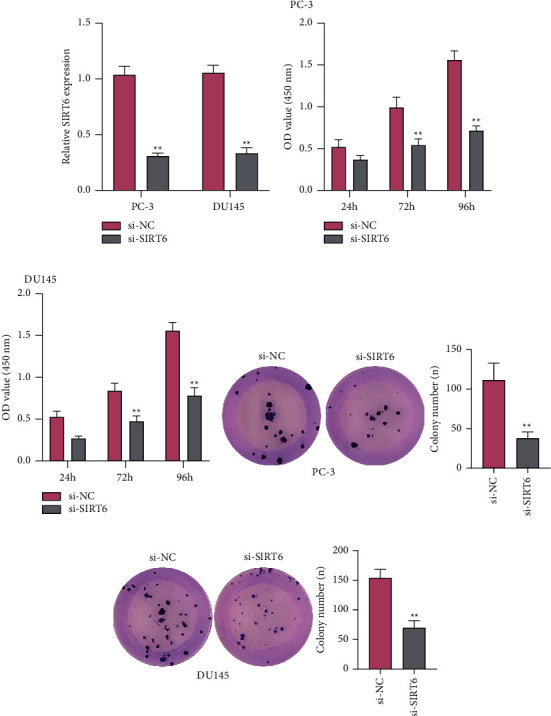
Knockdown of SIRT6 suppressed the proliferation of PC-3 or DU145 cells. (a) qPCR assays detected the expression levels of SIRT6 in PCa cells after transfected with si-SIRT6 or si-NC. (b, c) CCK-8 assays evaluated the cellular growth after treatment with si-SIRT6 or si-NC at 48 h, 72 h, and 96 h. (d and e) Colony formation assays assessed the effects of SIRT6 knockdown on the clonogenic capacity of PCa cells. ^*∗∗*^*p* < 0.01.

Because SIRT6 was lowly expressed in PCa, we next conducted gain-of-function studies using si-NC transfection to examine the functions of SIRT6 in PCa. The results of qPCR suggested the distinct overexpression of SIRT6 in PC-3 or DU145 cells (Figure 2(a)). CCK8 assays were then carried out to evaluate the potential biological roles of SIRT6 in PCa cell proliferation. After transfecting si-SIRT6, the cellular growth of PCa cells was significantly decreased at 72 h and 96 h (Figures 2(b) and 2(c)). In addition, the colony formation assays demonstrated that the silence of SIRT6 distinctly suppressed the clonogenic abilities of PCa cells (Figures 2(d) and 2(e)).

### 2.3. Effects of SIRT6 on the Migration and Invasion of PCa Cells

**Figure 3:**
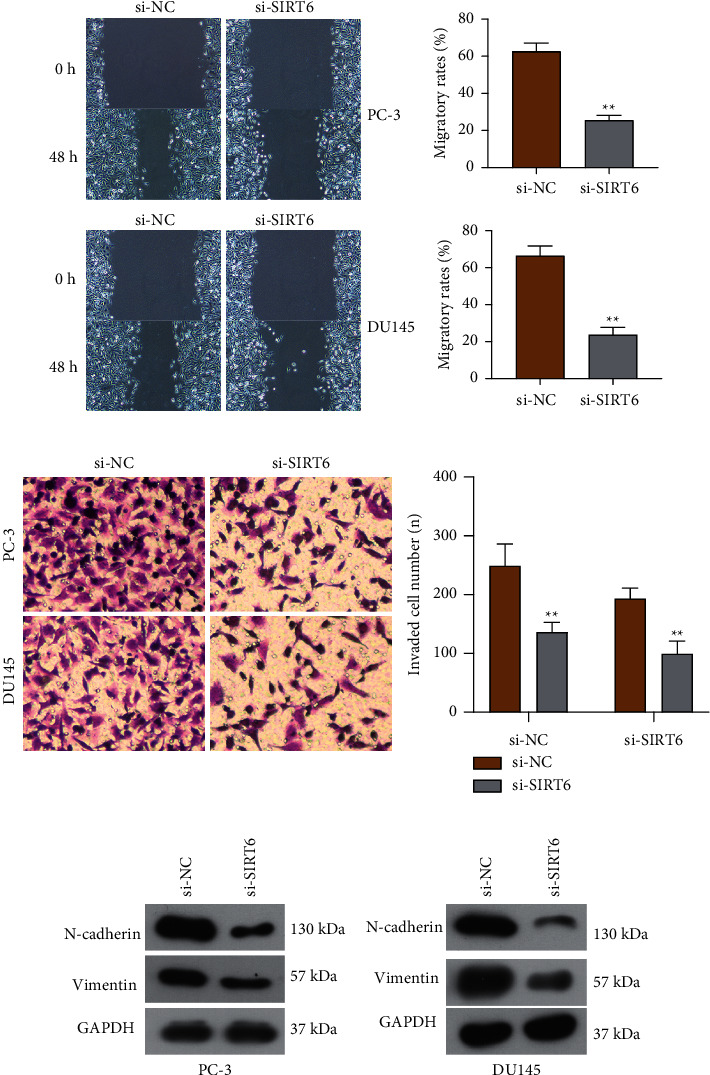
Knockdown of SIRT6 impaired the invasion and migration of PCa cells. (a) Migration was inhibited in PCa cells when transfecting with si-NC or si-SIRT6. (b) Transfection of si-SIRT6 reduced the invasion of PCa cells. (c, d) Western blot measured the protein expressions of N-cadherin and vimentin. The relative optical density of the protein bands was analyzed by Image J software (NIH, Bethesda, MD, USA). ^*∗*^*p* < 0.05; ^*∗∗*^*p* < 0.01.

To further explore the roles of SIRT6 in the migration and invasiveness of PCa cells, we conducted wound healing and transwell invasion assays using PC-3 or DU145 cells after treatment of si-SIRT6. The data of wound healing assays validated that knockdown of SIRT6 dramatically suppressed the width of wounded areas (Figure 3(a)). Furthermore, with the downregulation of SIRT6, the invasive capability of PCa cells was notably reduced when they were assessed by transwell invasion assays (Figure 3(b)). In addition, to elucidate the mechanisms of SIRT6 on cell metastasis, we carried out Western blot analysis to evaluate the levels of N-cadherin and vimentin which were involved in epithelial-mesenchymal transition. The data demonstrated that silence of SIRT6 led to obvious decline of N-cadherin and vimentin protein levels in PCa cells (Figures 3(c) and 3(d)). Collectively, these data provided evidence that SIRT6 served as an important regulator in modulating the migration and invasion of PCa cells.

### 2.4. Depression of SIRT6 Impeded the Activation of Wnt/*β*‐Catenin Signaling in PCa Cells

**Figure 4:**
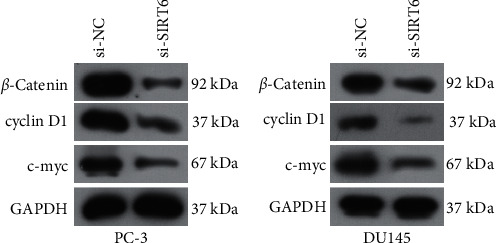
The activity of Wnt/*β*‐catenin signaling in PCa cells was depressed by SIRT6 silence. The protein levels of c-myc, cyclin D1, and *β*-Catenin in PC-3 (a) and DU145 (B) cells were decreased after SIRT6 silence.

We next aimed to ascertain the detailed mechanisms by which SIRT6 orchestrated cellular ability. Wnt/*β*‐catenin signaling, a well-known signaling which was closely associated with the functional regulation of multiple cancers, was investigated in the following experiments [19]. Hence, Western blot assays were utilized to evaluate the protein levels of molecules involved in Wnt/*β*‐catenin signaling. The results indicated that the protein levels of c-myc, cyclin D1, and *β*-catenin were remarkably decreased in PC-3 and DU145 cells (Figures 4(a) and 4(b)). Overall, these data revealed that the activation of Wnt/*β*‐catenin signaling was suppressed by SIRT6 knockdown in PCa cells, and our above results implied that SIRT6 modulated the development and progression of PCa via affecting Wnt/*β*‐catenin signaling.

## 3. Discussion

To date, many PCa patients with advanced stages have an unfavorable clinical outcome because of limited chemotherapy and the antibiotic drugs [20]. Thus, the identification of novel therapeutic targets is very important for the clinical outcome of PCa patients. Here, our group observed that SIRT6 expression was distinctly increased in PCa specimens compared with nontumor specimens. Then, the knockdown of SIRT6 was shown to suppress the proliferation, migration, and invasion of PCa cells, indicating its oncogenic roles in PCa progression. Previously, several studies reported the dysregulation of SIRT6 in several types of tumors [16, 21]. For instance, SIRT6 was shown to be lowly expressed in colorectal cancer stem cells, and its overexpression suppressed the cell proliferation, colony formation, and induced G0/G1 phase arrest in colorectal cancer stem cells via regulating CDC25A [22]. However, high levels of SIRT6 were observed in diffuse large B-cell lymphoma, and its overexpression promoted the metastasizing capacity of tumor cells and drug resistance of diffuse large B-cell lymphoma by mediating PI3K/Akt signaling [17]. These findings suggested a different role of SIRT6 based on the types of tumors. Our findings indicated SIRT6 as an oncogene, which was not consistent with its function in breast cancer and lung cancer [23, 24].

Epithelial-mesenchymal transition (EMT) is a process in which epithelial cells acquire mesenchymal features [25]. It has been demonstrated that EMT is involved in cancer progression, metastatic competency, and problems of drug resistance [26, 27]. In recent years, more and more studies have shown that some tumor-related genes displayed their oncogenic or antioncogenic functions on tumor progression via modulating the EMT pathway [28, 29]. In this study, we also observed that SIRT6 knockdown distinctly suppressed EMT progress. Previously, SIRT6 was reported to promote an aggressive phenotype and the EMT in papillary thyroid cancer, which was consistent with our findings [30]. However, more experiments were needed to further explore the possible effects of SIRT6 on EMT progress.

Wnt/*β*‐catenin signaling is evolutionarily conserved and required for embryonic development and tissue homeostasis [31]. Wnt/*β*‐catenin signaling is frequently reported to participate in the development and progressions of various types of tumors [32, 33]. This signaling pathway is highly conserved throughout evolution, and it is important in intercellular communication [34]. Growing evidence indicate that enhancing Wnt/*β*‐catenin signaling elements' expression, like receptors and downstream targets, is important in overcoming drug resistance and the reversal of the EMT phenotype [35, 36]. In this study, PCa cells were transduced with si-SIRT6, and we found that the protein level of c-myc, cyclin D1, and *β*-Catenin were remarkably decreased, suggesting this pathway was inversely modulated by SIRT6 in PCa cells. Thus, our findings indicated that silence of SIRT6 suppressed PCa progression via modulating Wnt/*β*‐catenin signaling.

## 4. Conclusions

Our findings provided novel evidence that SIRT6 was highly expressed in PCa and promoted the proliferation and metastasis of PCa cells. Mechanistically, SIRT6 silence suppressed the activity of Wnt/*β*‐catenin signaling. Together, our findings suggested that SIRT6 could become a novel prognostic biomarker and potential therapeutic target in PCa.

## Data Availability

The data sets generated during and/or analyzed during the current study are available from the corresponding author upon reasonable request.
